# Supplementary material for: Effect of a decision aid on the choice of pregnant women whether to have epidural anesthesia or not during labor
Source: PLoS One. 2020 Nov 12;15(11):e0242351. doi: 10.1371/journal.pone.0242351 (PMC7660548; doi:10.1371/journal.pone.0242351)
Supplement: Attachment — Submitted filename: reviewPONE-D-20-18281.pdf [file pone.0242351.s002.pdf]

Dear Editor,

I first would like to thank you for giving me the opportunity to review this original manuscript intended for publication in PlosOne.

The authors aim through their study to compare the effect of a decision aid (DA) on pregnant women's decision on using epidural anesthesia or not for labor during vaginal delivery.

Please find hereby various comments:

In the abstract, it is not clearly stated the rational of the study, why should we conduct such study ?

In the abstract, presentation of some results are in conclusions, when new data shouldn't be introduced in there.

In the abstract, there is no results with numbers. We have to "trust" the author as they don't show any statistics and numerical results.

In study design please be more specific about the "contraindication": regarding epidural ? regarding … ?

Please specify number of deliveries per year in the hospital where the research took place.

Line 134: study protocol. End of study is after delivery ?

How was the 300 patients limit defined. Is the power of the study sufficient to bring results ?

At the end of the study, each group were given the other group's documentation. Was it evaluated if after that they would have changed their mind regarding the decision ?

Line 169: Alpha testing. Please specify number of people in each group of experts.

Line 179-182: Not clear to which criteria/scale it is each time referring to.

Line 279: please introduce this review in intro, as you base most of your comparison in the discussion with this paper.

Line 332 : your scale has not been validated by other studies or group is that correct ? Plus, limitation to Japanese women should be stated, as access to analgesia and representation of pain management varies from one country to another.

How can you be sure that your DA does not influence the patient in choosing epidural over no analgesia (figure 1a : 60->80 patients chose epidural) , when the aim is just to provide general information.

Bias not assessed: previous painfull delivery. Previous Epidural anesthesia for delivery or other previous surgery.

It is not clear for me when each test was conducted, from 34weeks until post-partum. Is it possible to have a schematic timeline to place each test ?

It is not clear, from the 300 patients, how many did effectively deliver with/without epidural. From which group respectively. (possibly, provide a tree diagram)

It is not clear for me whether pre-labour decision was followed by effective choice for epidural or not, for each group. Meaning: the parturient can be happy with her choice or not, but can be happy to have changed her mind.

It is not clear for me when the test for knowledge of epidural was conducted. Before the DA information? Was there a comparison of the knowledge before and after the intervention and after the delivery ?

Q: The uncertainty group had a lower DCS, but because they chose Epidural or not ?

Ad a final Summary figure or flowchart / algorithm for clinical use

Short resumé of the main question asked and the main answer

1. Is the research question worth asking? YES
2. What does this paper bring new ? ASSESSMENT OF PATIENTS SATISFACTION AND AID TO DECISION
3. Is there already publications about this subject ? YES
4. What is the actual evidence ? POOR
5. Is this paper going in the same trend ? YES
6. Do they answer the question of their title ? YES
7. Are the methods appropriate and sufficiently robust to answer the stated research question? MODERATELY
8. Is the patient population sufficiently representative? UNKNOWN
9. Are the groups appropriately balanced to minimize confounders? POOR SOCIO-DEMOGRAPHIC INFORMATIONS
10. Were the groups treated equally and steps taken to minimize bias at all stages of the study? YES
11. Are the outcomes suitably objective? YES, BUT COULD BE EXPLANNED MORE CLEARLY
12. Is the follow-up period sufficiently long? NA, BUT COULD BE PART OF AN OTHER FOLLOW UP STUDY
13. Are the limitations of the work discussed? Critical discussion about their weak point on the study ? COULD BE ENHANCED

14. Are the conclusions supported by the data and the methods? YES, BUT SHOULD BE MADE EASIER TO READ
15. Does this manuscript truly add to the literature in a meaningful way? *Nothing really new but adds to the existing body of evidence*
16. . Have they provided sufficient methodological detail that the experiments could be reproduced? YES
17. Is the statistical analysis of the data sound? NO
18. Overall clarity of the presentation and flow of logic? PLEASE READ COMMENTS
19. Is the manuscript clearly written? PLEASE READ COMMENTS
20. Please give this manuscript an overall score out of 100. 70
21. Did they split the data to allow multiple publications? UNKNOWN
22. Is there major methodological flaws? NO
23. Are there any special ethical concerns arising from the use of animals or human subjects? NO
24. Used abbreviations in the abstract, Publication, paragraph of correct length ? Yes
25. English quality ? GOOD
26. Did everybody on the author list contribute on the publication ? AT LEAST AS STATED SO BY THE AUTHORS.

	Yes	Can be improved	Must be improved	Not applicable
Does the introduction provide sufficient background and include all relevant references?	()	( )	(X)	( )
Is the research design appropriate?	(X)	( )	( )	( )
Are the methods adequately described?	()	( )	(X)	( )
Are the results clearly presented?	(x)	( )	( )	( )
Are the conclusions supported by the results?	(x)	( )	( )	( )
