# Supplementary material for: Effect of a decision aid on the choice of pregnant women whether to have epidural anesthesia or not during labor
Source: PLoS One. 2020 Nov 12;15(11):e0242351. doi: 10.1371/journal.pone.0242351 (PMC7660548; doi:10.1371/journal.pone.0242351)
Supplement: Attachment — Submitted filename: Plos_One_decision_aid_anesthesia.docx [file pone.0242351.s003.docx]

**Reviewing: Plos One PONE-D-20-18281**

**“Effect of a decision aid on the choice of pregnant women whether to have epidural anesthesia or not during labor”.**

General comment:

The article shows data from a single-center non-randomized controlled trial taking place in Japan. The topic of this study is of interest. As described by the authors, few publications focus on decision aids on pregnant women although these are very interesting tools to promote shared-decision making process. Here are my comments throughout the manuscript:

Abstract

Please detail the number of women included in each group as well as the difference in means for each outcome.

Introduction

The introduction is globally clear. Some points could be clarified:

Compared to other developed country, the rate of epidural analgesia seems very low in Japan. Could you explain a little more these low rates and the reason for the low use of epidural analgesia (cultural? economic? organizational?). Precise in the method section the rate of epidural analgesia in your birth centre.

It seems that you consider negatively being ‘undecided’ or in ‘decision conflict’ for pregnant women regarding their wish to have or not epidural analgesia. Is there any data to support this point (lower satisfaction, increased anxiety, poorer outcomes? Etc.), in the context of epidural analgesia or more globally in studies dealing with shared-decision making? It makes sense that, even with the best level information and knowledge, some women, especially primiparous who have never experienced such level of pain, remained undecided about analgesia until delivery, because they cannot anticipate their wish or not have analgesia.

Methods

Please be more precise, if possible, when describing how women receive information about epidural analgesia: who give the pamphlet or DA (midwife or anaesthetist) and is the document is completed by an oral information? Is it possible that the information given (apart from the document) was different between the 2 periods?

To get an idea of the reading effort for pregnant women, you may detail le time required to read each document in Japanese (pamphlet and DA).

Do the DA describe alternative pain management methods?

Why did you choose to measure DSC and satisfaction at one week and not at or just after delivery?

Statistical analysis

Please precise how you define the study sample.

It is not clear in the statistical analysis part that the outcomes will be assessed by differences in score means. It should be specified.

Before performing Student t test, have you checked that hypothesizing a normal distribution seemed correct? Please consider justifying that somewhere.

Results and figure

A Flowchart should be added with the exact number of eligible women during the period and and the number of women non included with the reason. It could also be interesting, if possible, to present as additional data the comparison of eligible women included versus non included to document selection bias.

In addition to the differences of means, you should present the means score for the pre-test for each group to see if there was baseline difference. For example, how to explain that there seems to be a difference in the rate of indecision (Figure 1), which was more important at baseline in the control versus intervention group? Can we assume that the discourse of caregivers changed in intervention period?

Presenting these means would also allow us to know the mean level of knowledge, satisfaction and DCS. If the difference is significant but the overall level is low, the tools remain insufficient.

Figure 1: you should detail the percentage rather than the number for clarity.

Discussion

Please discuss why you couldn’t do a randomized controlled trial in the limit section of your manuscript. It could have prevented from comparability bias.

Have you checked that your results were not affected by the difference in educational level between control and intervention group by doing sensitivity analysis among high education level women? It may help you to discuss the potential selection bias.
