# Supplementary material for: Effect of a decision aid on the choice of pregnant women whether to have epidural anesthesia or not during labor
Source: PLoS One. 2020 Nov 12;15(11):e0242351. doi: 10.1371/journal.pone.0242351 (PMC7660548; doi:10.1371/journal.pone.0242351)
Supplement: Attachment — Submitted filename: Response to Reviewers.docx [file pone.0242351.s004.docx]

October 8, 2020

**David Desseauve**

Academic Editor
*PLOS ONE*

**Subject**: Submission of revised manuscript (PONE-D-20-18281) entitled, “**Effect of a decision aid on the choice of pregnant women whether to have epidural anesthesia or not during labor**” by Shishido et al.

Dear Dr. Desseauve,

Thank you for your careful review and valuable suggestions regarding our manuscript. We greatly appreciate the constructive comments by the reviewers which have helped us to considerably improve our paper.

We have thoroughly revised our manuscript in accordance with all the reviewers’ comments. All revisions and newly added text in the revised manuscript are indicated by *track changes* in accordance with the resubmission instructions. We have also provided below our *point-by-point responses* to each of the comments raised.

The revised manuscript has also been comprehensively reviewed and edited for English language by a professional native English-speaking medical and nursing science editor to meet the language standards required by leading English-language publications. The editing did not involve any alteration of the research content or the authors’ intentions.

We hope that we have satisfactorily answered all the concerns of the reviewers, and that the revised manuscript is now acceptable for publication in *PLOS ONE*.

Thank you for considering our revised manuscript. We look forward to hearing from you at your earliest convenience.

Sincerely yours,

**Eri Shishido, PhD, MN, CNM**

St. Luke’s International University

10-1 Akashi-cho, Chuo-ku, Tokyo104-0044, Japan

Phone: +81-3-3543-6391

Fax: +81-3-5565-1626

E-mail: 17DN003@slcn.ac.jp

**RESPONSES TO REVIEWERS’ COMMENTS**

**Comments from the reviewers:**

**Reviewer #1**

**Thanks to editorial committee for the opportunity to review this work. The paper aim to investigate the effect of a decision aid tool for women in order to help them to make a decision between planned epidural analgesia and planned no epidural analgesia. The authors hypothesize that a specific decision aid tool may reduce the part of prenatal undecided women. The paper is well written original. There are some limitations that should be addressed in a revised version. It is unclear why the authors did not planned a randomized trial, a power calculation is missing, the assignation procedure into a group is unclear, there is no evaluation of postnatal satisfaction about pain management.**

**Response**:

We wish to express our deep appreciation to you for recognizing the importance of our study. Thank you for your favorable appraisal of the originality of the relevant data obtained. We also appreciate your valuable comments on the study design and limitations. We have responded to the main points that you mentioned in our point-by-point responses below. Thank you for the opportunity to clarify these points and make the necessary changes in the revised manuscript in accordance with your comments.

**Comment 1)
Abstract
The first paragraph is wordy “decision aid on pregnant women’s decisions….”**

**- The objective reported here is non informative, we don’t know what is the main analysis**

**Response:**

Thank you for your important comments. We carefully revised the first paragraph of the abstract to make it clear and concise. We also specified the objective and indicated what the primary outcome is in the revised manuscript and as shown below (Page 2, Lines 21-25 of the revised manuscript without the track changes):

“Decision aids (DAs) are useful in providing information for decision-making on using epidural anesthesia during birth. To date, there has been little development of DAs for Japanese pregnant women. Herein, we investigated the effect of a DA on the decision of pregnant whether to have epidural anesthesia or not for labor during vaginal delivery. The primary outcome is changes in mean decision conflict score.”

**Comment 2)
Please report, at least, the number of included women and indicate how women were assigned into the intervention group or not.**

**Response:**

We appreciate your important suggestion. We described the number of women included in each group and indicated how many women were assigned into each group as follows (Page 2 Lines 27-30):

“In this non-randomized controlled trial, 300 low-risk pregnant women in an urban hospital were recruited by purposive sampling and assigned to 2 groups: DA (intervention) and pamphlet (control) groups. Control enrollment was started first (until 150 women), followed by intervention enrollment (150 women).”

**Comment 3)
Considering that the main objective and the main judgement criterion are not clearly defined, conclusions appears not interpretable**

**Response:**

We have clearly defined the main objective and primary outcome of the study in the abstract, and revised the conclusion making it interpretable in reference to the main objective as follows:

(Page 2, Lines 23-25 Objective)

“Herein, we investigated the effect of a DA on the decision of pregnant whether to have epidural anesthesia or not for labor during vaginal delivery. The primary outcome was changes in mean decision conflict score.”

(Page 3, Lines 46-47 Conclusion)

“This study indicates that a DA can be useful in helping women make a decision whether to have epidural anesthesia or not for labor during vaginal delivery.”

**Comment 4)
Introduction
- Consider providing data explaining why such a low rate of epidural analgesia compared to other countries like France with more than 50% of woman benefiting from epidural analgesia.**

**Response:**

Thank you for your important comment and advice. Indeed, the low rate of epidural analgesia in Japan compared with other countries is evident. This is attributable to various factors which we additionally explained in the revised manuscript as follows (Page 3, Line 52 to Page 4, Line 58):

“In Japan, the proportion of women receiving epidural anesthesia increased from 2.6% in 2008 to 6.2% in 2016 [1, 2]. However, compared with other countries, the rate of analgesic delivery in Japan is low. This is because of the small percentage of hospitals that can provide epidural anesthesia (15.0%, 2017), failure to provide information about epidural anesthesia to all pregnant women, and a cultural belief that labor pain is a virtue [3, 4]. In Japan, about half of deliveries are performed in hospitals and clinics. As there are no anesthesiologists in all facilities, only a few are able to perform epidural anesthesia.”

**Comment 5)**

**The review of literature should be condensed for reducing the volume of the introduction part which is too long**

**Response:**

Thank you for your important advice. We have condensed the review of literature to reduce the volume of the Introduction section in the revised manuscript (Pages 3-6).

**Comment 6)**

**Lines 70-74: what about information delivered by anesthesiologists?**

**Response:**

Regarding information delivered by anesthesiologists, we added an explanation in the revised manuscript as follows (Page 5, Lines 74-75):

“The anesthesiologist provides information on the indications, contraindications, and benefits/risks of anesthesia in the outpatient setting.”

**Comment 7)
In the last paragraph, the main objective and related and judgement criterion remains unclear to me. This should be clarified. Is that as follows: investigating the effect of a DA on prenatal women’s decision about epidural analgesia use; with / without / undecided. If it is you may consider formulate an hypothesis, meaning that you hypothesis that DA increase the rate prenatal epidural wishes.**

**Response:**

We truly appreciate your valuable comments and suggestion. Based on your advice, we carefully clarified both the main objective and the judgement criterion in the Introduction section as follows (Page 6, Lines 96-98):

“Specifically, this study aimed to investigate the effect of a DA on pregnant women’s decision whether to use epidural anesthesia or not when planning to have vaginal delivery. The primary outcome was changes in the mean decision conflict score.”

In accordance with your suggestion, we formulated and added our hypotheses regarding the effects of using a DA as follows (Page 6, Line 99 to Page 7, Line 111):

“**Hypotheses**

Our main hypothesis is that the utilization of a DA as intervention is useful in helping prenatal women decide on the use of epidural anesthesia when planning for vaginal delivery. Our specific hypotheses are as follows:

1. The mean decision conflict score is reduced in the intervention group compared with the control group.

2. The mean satisfaction with decision-making score after the intervention is higher in the intervention group than in the control group.

3. The mean knowledge score of epidural anesthesia is higher in the intervention group than in the control group.

4. The proportion of pregnant women who are undecided whether to deliver with or without epidural anesthesia is decreased in the intervention group compared with the control group.”

**Comment 8)
Methods**
**Justify why not implement a randomized trial**

**Response:**

Thank you for your important comment. To our knowledge, there is still no study in Japan with regards to the use of a DA for assisting informed decision-making whether to use epidural anesthesia or not. The reasons vary from the small percentage of hospitals that can provide epidural anesthesia, failure to provide information about epidural anesthesia to all pregnant women, to a cultural belief in Japan that labor pain is a virtue. To the best of our knowledge, ours is the first study to do so. However, at the outset, we had existing limitations such that one of the authors belonged to a recruited hospital, there were missing educational data primarily from the control group, difficulty in ascertaining the knowledge gained, and the inclusion of both nulliparous and parous women. These hindered the rigorous performance of an exact and strict RCT or would have resulted in an RCT with a number of biases that would have affected the outcomes. We assessed that an **nRCT** at this stage would be sufficient as a pioneering foundation for a subsequent RCT devoid of selection bias in previous parity or delivery or other limitations. We have added an explanation regarding these points in the revised manuscript with an important note for planning an RCT in the future (Page 24, Lines 396-407):

“A limitation in this study is that it is not known if the 2 groups were similar because of missing educational data, which was primarily from the control group. Also, we were not able to ascertain the knowledge gained by the participants from other sources of information (e.g., books, internet) between the pre-test and the post-test. It is important to state that the subjects of this study were limited to Japanese women, as access to analgesia and pain relief differs by country, culture, and medical system. As this study was not an RCT, bias in the results cannot be completely ruled out. A subsequent RCT is necessary to allow generalization of the results. In the future, we need to consider against selection bias in parity and previous surgery/delivery. An assessment as to how a rigorous RCT could be performed is underway as the DA is being planned to be uploaded and viewed on the web rather than in paper form.”

**Comment 9)**

**It seems that you included both nulliparous and parous women. Don’t you think that it could biases your analysis?**

**Response:**

Thank you for your valuable comment. We understand your concern and thus acknowledged in the revised manuscript the possibility of selection bias due to obstetric history. We clarified such possibility of bias in the Limitations section and noted that the present work was not an RCT as follows (Page 24, Lines 402-407):

“As this study was not an RCT, bias in the results cannot be completely ruled out. A subsequent RCT is necessary to allow generalization of the results. In the future, we need to consider against selection bias in parity and previous surgery/delivery. An assessment as to how a rigorous RCT could be performed is underway as the DA is being planned to be uploaded and viewed on the web rather than in paper form.”

**Comment 10)
There is no justification for including 300 women. Your research question and the design requires an a priori power calculation. Formulate a hypothesis and, based on it, a power calculation. This approach is strongly limited by the fact that it is an a posteriori calculation and may be discussed below.**

**Response:**

We greatly appreciate your valuable comments. We added an explanation regarding the sample size and power calculation as follows (Page 7-8, Lines 125-130):

“**Sample size**

Stacey et al. and Raynes-Greenow reported that the intervention is effective if there is a difference in the conflict scores of -4.35 (95% CI -6.8 to -1.9) [10, 11] with a calculated 141 people needed in each group. In the present study, we also considered a dropout rate of about 10% and thus set the number of participants to 150 in each group for a total of 300 participants recruited (effect size 0.5; power 0.8; significance level 5%).”

**Comment 11)**

**How women were assigned to a group? This must be clearly reported**

**Response:**

With regard to assigning the women to a group, we started collecting data for the control group first. After reaching 150 women, we then started collecting data for the intervention group. We clearly explained this process in the study protocol as follows (Page 8, Lines 133-137):

“Participants were selected using purposive sampling. Enrollment for the control group was started first and this continued until 150 women who met the inclusion criteria agreed to participate and whose data were collected. Recruitment, enrollment, and data collection for the intervention group were conducted after the recruitment for the control group and followed the same protocol.”

We also added an explanation and a figure regarding the collection of data as follows (Page 8, Line 138-140):

“Data allocation for the control group was performed first from June 1 to July 10, 2019 (**Fig　1**). Data allocation for the intervention group was performed from July 15 to August 27, 2019. Data were collected from June 2019 to the end of August 2019.”

**Comment 12)**

**Finally you compared a 22 pages pamphlet versus a 11 pages pamphlet…. By reading this it seems to me that you compared the effect of two information pamphlet about analgesia during labor on women’s decision and not really a decision aid tool versus not…I mean here that both might be considered as decision aid tools with one more detailed than the other.**

**Response:**

Thank you for providing your valuable impression. There were differences between the DA and the pamphlet. The pamphlet only described in writing the benefits and risks of epidural anesthesia. The content is purely informational and does not include a decision-making component. On the other hand, the DA included the specific contents regarding epidural anesthesia and no epidural anesthesia, comparative tables of each option for making one’s own decision, values clarification exercise, and the decision-making process. We have originally stated these differences and how these materials were used in the manuscript. We added a few more lines to explain the specific differences as follows (Page 9, Lines 161 to Page 10, Line171):

“The DA (for the intervention group) and standard information pamphlet (for the control group) showed differences in the following items: 1) information on epidural anesthesia or no epidural anesthesia options, 2) comparative tables of each option, 3) values clarification exercise, and 4) decision-making process. The pamphlet only described in writing the benefits and risks of epidural anesthesia. The content is purely informational and does not include a decision-making component found in the DA.

These DA and pamphlet were to be read before the next antenatal check-up. At the next check-up about a week later, after confirming that the participants had read the pamphlet or DA, a post-test containing the same measurements was conducted. At the end of the post-test, the intervention group was given the pamphlet and the control group was given the DA.”

**Comment 13)
Please clearly expose your main objective being concordant with the primary outcome definition**

**Response:**

In accordance with your important advice, we clearly explained the main objective of the study in concordance with the primary outcome definition as follows (Page 6, Lines 96-98):

“Specifically, this study aimed to investigate the effect of a DA on pregnant women’s decision whether to use epidural anesthesia or not when planning to have vaginal delivery. The primary outcome was changes in the mean decision conflict score.”

**Comment 14)**

**Results
The difference about educational level between your 2 groups affect the validity of the results. We need a justification for not implementing a randomized trial and a more detailed description of the procedure allocation to a group.**

**Response:**

We appreciate your important advice. Similarly to our response to your comment 8), there is still apparently no study in Japan regarding the use of a DA for assisting informed decision-making on epidural anesthesia usage. The small percentage of hospitals that can provide epidural anesthesia, failure to provide information about epidural anesthesia to all pregnant women, and the cultural belief that labor pain is a virtue are contributing reasons for this lack of DA usage in Japan.

Our study at its outset had existing limitations such that one of the authors belonged to a recruited hospital, missing *educational data* primarily from the control group, difficulty in ascertaining knowledge gained, and the inclusion of both nulliparous and parous women. Although we found differences in the educational levels in this study, the results of the study were similar, although we excluded seven participants in the analysis. *These factors hindered the rigorous performance of an exact and strict RCT or would have resulted in an RCT with a number of biases that would have affected the outcomes*. We assessed that an **n**RCT at this stage would be sufficient as a pioneering foundation for a subsequent RCT devoid of selection bias. We have acknowledged these limitations and carefully explained them in the revised manuscript with an indication of a planned RCT in the future as follows (Page 24, Lines 396-407):

“A limitation in this study is that it is not known if the 2 groups were similar because of missing educational data, which was primarily from the control group. Also, we were not able to ascertain the knowledge gained by the participants from other sources of information (e.g., books, internet) between the pre-test and the post-test. It is important to state that the subjects of this study were limited to Japanese women, as access to analgesia and pain relief differs by country, culture, and medical system. As this study was not an RCT, bias in the results cannot be completely ruled out. A subsequent RCT is necessary to allow generalization of the results. In the future, we need to consider against selection bias in parity and previous surgery/delivery. An assessment as to how a rigorous RCT could be performed is underway as the DA is being planned to be uploaded and viewed on the web rather than in paper form.”

We also added *a more detailed description of the allocation procedure to each group* as follows (Page 8, Lines 133-137):

“Participants were selected using purposive sampling. Enrollment for the control group was started first and this continued until 150 women who met the inclusion criteria agreed to participate and whose data were collected. Recruitment, enrollment, and data collection for the intervention group were conducted after the recruitment for the control group and followed the same protocol.”

**Comment 15)
Discussion
In my opinion the key message of the paper is the reduction of undecided women and this point deserve to more highlighted in your paper**

**Response:**

We truly appreciate your valuable opinion. Indeed, the results showed that the DA was effective in helping undecided women with regard to their decision on epidural anesthesia usage. This was emphasized in the Results section as follows (Page 17, Lines 312-315):

“The number of pregnant women who were undecided whether to deliver with or without epidural anesthesia decreased significantly from 45 (30.2%) to 9 (6.1%) in the intervention group (p < .001) and only from 61 (40.7%) to 58 (38.9%) in the control group.”

These results were similar to the systematic review results. The primary outcome of this study was reduced mean DCS score, so this outcome is preferred.

**Comment 16)
I think that the most important criteria would have been “postnatal women’s satisfaction about pain management”. Discuss the fact that the primary outcome may lack of clinical significance. Ok, it is great women are less undecided with your DA but if the postnatal satisfaction about pain management is extremely low ….the impact of your DA on women’s health is none. If a randomized trial is implemented, this point should be addressed.**

**Response:**

Thank you for your valuable comments. This study was conducted with a primary focus on pregnancy and it investigated specifically the effect of a DA on the decision-making process of women. In the future, we would like to conduct an RCT regarding postnatal women’s satisfaction about pain management.

**Reviewer #2**

**Although the subject studied depends on the country's practices, this study is original and conducted with a rigorous method. It is based on the use of several validated scales clearly presented and explained. The results can be extrapolated to other choice situations in perinatal care. Limitations are clearly stated.
The study emphasizes the importance of the quality of information and the tools to improve it.**

**Response:**

We greatly appreciate you for recognizing the importance of our study. Thank you for your favorable appraisal regarding the originality of its design and relevant data. We are also grateful to you for acknowledging the potential of our method as a decision-making tool in the future. Thank you very much for your in-depth and favorable assessment.

**Reviewer #3**

**I first would like to thank you for giving me the opportunity to review this original manuscript intended for publication in PlosOne.**

**The authors aim through their study to compare the effect of a decision aid (DA) on pregnant women’s decision on using epidural anesthesia or not for labor during vaginal delivery.**

**Response:**

We wish to express our great appreciation to you for your thorough review, insightful comments, and valuable suggestions regarding our paper. Our responses to each of your comments are shown below.

**Comment 1)**

**In the abstract, it is not clearly stated the rational of the study, why should we conduct such study?**

**Response:**

We appreciate your comment regarding the need to clearly state the rational and importance of conducting this study. We carefully revised the rational and specified the objective and primary outcome of the study as follows (Page 2, Lines 21-25 Objective)

“Decision aids (DAs) are useful in providing information for decision-making on using epidural anesthesia during birth. To date, there has been little development of DAs for Japanese pregnant women. Herein, we investigated the effect of a DA on the decision of pregnant whether to have epidural anesthesia or not for labor during vaginal delivery. The primary outcome was changes in mean decision conflict score.”

**Comment 2)**

**In the abstract, presentation of some results are in conclusions, when new data shouldn’t be introduced in there.**

**Response:**

Thank you for calling our attention regarding this important point. We carefully modified the abstract to achieve consistency from Objective to Conclusion, and ensured that no results are presented in the Conclusion part as follows (Page 3, Lines 46-47):

“**Conclusion**

This study indicates that a DA can be useful in helping women make a decision whether to have epidural anesthesia or not for labor during vaginal delivery.”

**Comment 3)**

**In the abstract, there is no results with numbers. We have to “trust” the author as they don’t show any statistics and numerical results.**

**Response:**

We appreciate your important comments and apologize for this oversight. In accordance with your comments, we revised the Results part and added numerical results with statistical analysis as follows (Page 2, Line 36 to Page 3, Line 44):

“**Results**

Women in the DA group (n = 149: 1 excluded because she did not return post-test questionnaire) had significantly lower DCS score than those in the pamphlet group (n = 150) (DA: -8.41 [SD 8.79] vs. pamphlet: -1.69 [SD 5.91], p < .001). Knowledge of epidural anesthesia and satisfaction with decision-making scores of women who used the DA were significantly higher than those of women who used the pamphlet (p < .001). Women in the DA group showed a significantly lower undecided rate than those in the pamphlet group. The number of undecided women in the DA group significantly decreased from 30.2% to 6.1% (p < .001), whereas that in the pamphlet group remained largely unchanged from 40.7% to 38.9%.”

**Comment 4)**

**In study design please be more specific about the “contraindication”: regarding epidural ? regarding … ?**

**Response:**

Thank you for your valuable advice. We added a specific explanation regarding “contraindications” as follows (Page 7, Lines 119-123 in Study design and participants):

“The exclusion criteria included pregnant women with (1) an obstetric complications history (e.g., cesarean section), (2) a medical history (e.g., mental disorder), (3) a fetal disease, and (4) contraindications (e.g., blood coagulation disorders, aortic stenosis, and hypertrophic cardiomyopathy).”

**Comment 5)**

**specify number of deliveries per year in the hospital where the research took place.**

**Response:**

In accordance with your advice, we specified the number of deliveries per year in the hospital in the study protocol as follows (Page 8, Lines 142-144):

“The hospital has about 2300 deliveries per year, and about 60% of these deliveries choose to have an epidural anesthesia. The hospital can perform an epidural anesthesia any time.”

**Comment 6)**

**Line 134: study protocol. End of study is after delivery?**

**Response:**

Thank you for clarifying this point. A pamphlet or DA was given to the participants when they had completed their responses to the post-test questionnaire, not after delivery. We clarified this point as follows (Page 10, Lines 167-171):

“These DA and pamphlet were to be read before the next antenatal check-up. At the next check-up about a week later, after confirming that the participants had read the pamphlet or DA, a post-test containing the same measurements was conducted. At the end of the post-test, the intervention group was given the pamphlet and the control group was given the DA.”

**Comment 7)**

**How was the 300 patients limit defined. Is the power of the study sufficient to bring results?**

**Response:**

We appreciate this important comment and query. We clarified how the sample size was calculated, specifically the 300 patient limit in the revised manuscript. This study was calculated at 80% power, which was sufficient as the sample met this. We provided a detailed explanation under the “Sample size” subsection that was newly added to the Materials and methods section as follows (Page 7, Lines 125 to Page 8, Line 130):

“**Sample size**

Stacey et al. and Raynes-Greenow reported that the intervention is effective if there is a difference in the conflict scores of -4.35 (95% CI -6.8 to -1.9) [10, 11] with a calculated 141 people needed in each group. In the present study, we also considered a dropout rate of about 10% and thus set the number of participants to 150 in each group for a total of 300 participants recruited (effect size 0.5; power 0.8; significance level 5%).”

**Comment 8)**

**At the end of the study, each group were given the other group’s documentation. Was it evaluated if after that they would have changed their mind regarding the decision?**

**Response:**

Thank you for your helpful query. Follow up and evaluation of the changes after the post-test were not conducted as these were not part of the objective of the present study. In future studies, we hope to investigate which type of delivery was ultimately chosen and the satisfaction with the choice made.

**Comment 9)**

**Line 169: Alpha testing. Please specify number of people in each group of experts.**

**Response:**

Thank you for your helpful advice. We specified the number of people in each group of experts as follows (Page 12, Lines 207-210):

“We completed the alpha testing by consulting with a perinatal physician (n = 1), anesthesiologist (n = 2), experienced midwives (n = 3), experts in decision-making (n =1), and women (n = 15) with childbirth experience.”

**Comment 10)**

**Line 179-182: Not clear to which criteria/scale it is each time referring to.**

**Response:**

We appreciate your calling our attention for the need to clarify the criteria that must be met to be classified as a DA. We carefully revised the description for ease of understanding as follows (Page 13, Lines 218-228):

“The development process and shared decision-making design components of the DA prototype were evaluated using the Japanese version of the International Patient Decision Aid Standards instrument (IPDASi version 4.0). The DA prototype met many quality standards of the Japanese version of IPDASi (version 4.0) developed by Osaka et al. (2017) [19]. Specifically, the DA prototype met all 6 *qualifying criteria* of the Japanese version of IPDASi (version 4.0) to be considered as a DA, that is, 6 out of 6 certification criteria after excluding 4 items that were not related to the examination for a high risk of harmful bias, and 14 out of 28 *quality criteria* considered to strengthen a DA but whose omission does not present a high risk of harmful bias. These 6 qualifying criteria should all be met to be classified as a DA, and meeting all the certification criteria indicates no harmful bias. Therefore, the quality of the DA is ensured.”

**Comment 11)**

**Line 279: please introduce this review in intro, as you base most of your comparison in the discussion with this paper.**

**Response:**

Thank you for your important advice. We introduced the systematic review of Stacey et al (Reference 10) in the Introduction section as follows (Page 5, Lines 83-85):

“In their systematic review, Stacey et al. reported that DAs reduced decision conflicts, increased decision satisfaction and knowledge, and decreased the proportion of participants who were unable to make decisions [10].”

**Comment 12)**

**Line 332: your scale has not been validated by other studies or group is that correct ? Plus, limitation to Japanese women should be stated, as access to analgesia and representation of pain management varies from one country to another.**

**Response:**

We appreciate your important comments. The Decision Conflict Scale (DCS) used for the primary outcome of this study has already been validated in previous studies (O’Connor, 1995; Kawaguchi et al., 2013; JinShil et al., 2017). We clarified this point in the revised manuscript as follows (Page 23, Lines 393-395):

“In this study, we used a reliable, previously confirmed, and valid scale (i.e., DCS) for the primary outcome, and a nonequivalent control group that controlled for internal validity [21, 26, 27].”

We also explained in the Limitations section that access to analgesia was limited to Japanese women and that there are differences in pain management between countries as follows (Page 24, Lines 400-402):

“It is important to state that the subjects of this study were limited to Japanese women, as access to analgesia and pain relief differs by country, culture, and medical system.”

**Comment 13)**

**How can you be sure that your DA does not influence the patient in choosing epidural over no analgesia (figure 1a: 60->80 patients chose epidural) , when the aim is just to provide general information.**

**Response:**

Thank you for your valuable comment. The DA provides information regarding each option objectively and without showing bias. It is possible that the women who were originally undecided were more interested in epidural anesthesia. We believe that it is important to get the right information and make one’s own decision whichever choice is better, as what occurred for each woman in this study.

**Comment 14)**

**Bias not assessed: previous painfull delivery. Previous Epidural anesthesia for delivery or other previous surgery.**

**Response:**

Thank you for your important comment. We mentioned in the Limitations section regarding the need to assess selection bias in future studies as follows (Page 24, lines 404-407):

“In the future, we need to consider against selection bias in parity and previous surgery/delivery.”

**Comment 15)**

**It is not clear for me when each test was conducted, from 34weeks until post-partum. Is it possible to have a schematic timeline to place each test?**

**Response:**

We appreciate your helpful suggestion. We prepared a new figure (**Figure 1**) indicating the timeline for placing each test (Page 10, line 173):

“**Figure 1.** Timeline of intervention procedure”

**Comment 16)**

**It is not clear, from the 300 patients, how many did effectively deliver with/without epidural. From which group respectively. (possibly, provide a tree diagram)**

**Response**:

Thank you for your valuable suggestion. We have created a diagram to show the flow of participants for the intervention and control groups **(Figure 2)**. This figure provides information regarding the allocation of patients to each group and who received the DA (intervention group) or pamphlet (control group). In this study, the primary outcome is the change in the mean DCS score. This means that the aim of the present study at this stage is to determine the changes in decision on the use of analgesia (i.e., without, undecided, or with epidural anesthesia). This study did not determine how many effectively delivered with or without epidural anesthesia for each group as this was outside its present scope and aim. The results of the changes in decision on using anesthesia are thus shown in Figure 3. The determination as to how many effectively delivered with or without epidural anesthesia for the Intervention and Control groups will be considered in a planned RCT in the future.

**Comment 17)**

**It is not clear for me whether pre-labour decision was followed by effective choice for epidural or not, for each group. Meaning: the parturient can be happy with her choice or not, but can be happy to have changed her mind.**

**Response:**

We appreciate your valuable comments and understand the various thoughts you have regarding the study. This study, however, only aimed to determine the changes in decision on the use of analgesia (i.e., without, undecided, or with epidural anesthesia). The aim of this study did not include an investigation into the actual method of delivery or satisfaction. We would like to examine these aspects in subsequent RCT studies.

**Comment 18)**

**It is not clear for me when the test for knowledge of epidural was conducted. Before the DA information? Was there a comparison of the knowledge before and after the intervention and after the delivery?**

**Response:**

Thank you for your important comments and queries. The test for knowledge of epidural anesthesia was conducted before and after the intervention. This is shown in the newly added **Figure 1**.

We also compared the knowledge of epidural anesthesia before and after the intervention as described below. A comparison of the knowledge of epidural anesthesia after delivery was not conducted as this was outside the aim of the present study.

(Page 2, Lines 38-40 Abstract)

“Knowledge of epidural anesthesia and satisfaction with decision-making scores of women who used the DA were significantly higher than those of women who used the pamphlet (p < .001).”

(Page 18, Line 307-309 Results)

“The pre-test showed that the intervention group had a significantly higher knowledge score (0-10) than the control group (intervention group: 7.34 [SD 1.20] vs. control group: 6.59 [SD 1.33], p < .001). The mean score of knowledge of epidural anesthesia was significantly higher in women assigned to the intervention group than in women assigned to the control group (intervention group: 1.96 [SD 1.63] vs. control group: 1.33 [SD 1.44], p <.001).”

(Page 19 Table 3)

Table 3 shows a comparison of the mean score differences in the secondary outcomes (i.e., Satisfaction with decision-making and Knowledge of epidural anesthesia) from the pre-test to the post-test in the Intervention and Control groups.

**Comment 19)**

**The uncertainty group had a lower DCS, but because they chose Epidural or not ?**

**Ad a final Summary figure or flowchart / algorithm for clinical use**

**Response:**

Thank you for your valuable comment. Previous studies have shown that pregnant women who are undecided

about epidural anesthesia have a lack of knowledge and unknowns regarding childbirth (Shishido et al., 2018). We presume that there was a conflict because of these. In the future, we hope to consider algorithms that can be used in clinical practice.

**Reviewer #4**

**The article shows data from a single-center non-randomized controlled trial taking place in Japan. The topic of this study is of interest. As described by the authors, few publications focus on decision aids on pregnant women although these are very interesting tools to promote shared-decision making process.**

**Response:**

We wish to express our great appreciation to you for your insightful comments and valuable suggestions regarding our paper. Your important comments and advice have helped us to significantly improve our paper. Our point-by-point responses to your comments are shown below.

**Comment 1)**

**Abstract**

**Please detail the number of women included in each group as well as the difference in means for each outcome.**

**Response:**

We appreciate your important advice regarding the inclusion of details. We described the number of women in each group and added the primary outcome in the Results as follows (Page 2, Line 36 to Page 3, Line 44):

“Women in the DA group (n = 149: 1 excluded because she did not return post-test questionnaire) had significantly lower DCS score than those in the pamphlet group (n = 150) (DA: -8.41 [SD 8.79] vs. pamphlet: -1.69 [SD 5.91], p < .001). Knowledge of epidural anesthesia and satisfaction with decision-making scores of women who used the DA were significantly higher than those of women who used the pamphlet (p < .001). Women in the DA group showed a significantly lower undecided rate than those in the pamphlet group. The number of undecided women in the DA group significantly decreased from 30.2% to 6.1% (p < .001), whereas that in the pamphlet group remained largely unchanged from 40.7% to 38.9%.”

**Comment 2)**

**Introduction**

**The introduction is globally clear. Some points could be clarified:**

**Compared to other developed country, the rate of epidural analgesia seems very low in Japan. Could you explain a little more these low rates and the reason for the low use of epidural analgesia (cultural? economic? organizational?).**

**Response:**

We appreciate your important comments and advice. We added an explanation about the low rates of use of epidural analgesia in Japan as follows (Page 3, Line 52 to Page 4, Line 58):

“In Japan, the proportion of women receiving epidural anesthesia increased from 2.6% in 2008 to 6.2% in 2016 [1, 2]. However, compared with other countries, the rate of analgesic delivery in Japan is low. This is because of the small percentage of hospitals that can provide epidural anesthesia (15.0%, 2017), failure to provide information about epidural anesthesia to all pregnant women, and a cultural belief that labor pain is a virtue [3, 4]. In Japan, about half of deliveries are performed in hospitals and clinics. As there are no obstetricians or anesthesiologists in all facilities, only a few are able to perform epidural anesthesia.”

**Comment 3)**

**Precise in the method section the rate of epidural analgesia in your birth centre.**

**Response:**

In accordance with your comment, we added the rate of epidural analgesia in our birth center under the Study protocol subsection of the Materials and methods section as follows (Page 8, Lines 142-144):

“The hospital has about 2,300 deliveries per year, and about 60% of these deliveries choose to have an epidural anesthesia. The hospital can perform an epidural anesthesia any time.”

**Comment 4)**

**It seems that you consider negatively being ‘undecided’ or in ‘decision conflict’ for pregnant women regarding their wish to have or not epidural analgesia. Is there any data to support this point (lower satisfaction, increased anxiety, poorer outcomes? Etc.), in the context of epidural analgesia or more globally in studies dealing with shared-decision making? It makes sense that, even with the best level information and knowledge, some women, especially primiparous who have never experienced such level of pain, remained undecided about analgesia until delivery, because they cannot anticipate their wish or not have analgesia.**

**Response:**

Thank you for your in-depth comments and valuable insights. We also recognize that some pregnant women are unable to decide due to unknown factors. For this reason, we added an explanation based on a previous research to support the results as follows (Page 22, Line 380 to Page 26, Line 388):

“In 1994, Brown and Lumley [13] described the satisfaction of 790 Australian women with their care at birth within the first week of delivery. They reported that the lack of involvement in decision-making and the lack of adequate information had a significant impact on the dissatisfaction of the women at the time of their delivery. In a focus group study, the women were reportedly unable to describe the risks and benefits of labor analgesia even though they had considered themselves to be knowledgeable about the topic [25]. It was suggested that the right information and making your own decisions during pregnancy could lead to delivery satisfaction.”

**Comment 5)**

**Methods**

**Please be more precise, if possible, when describing how women receive information about epidural analgesia: who give the pamphlet or DA (midwife or anaesthetist) and is the document is completed by an oral information**?

**Response:**

In accordance with your valuable comment and query, we described how the women received information about epidural analgesia as follows (Page 9, Line 145-19):

“When eligible pregnant women at 34 weeks of gestation visited for their maternity check-up, the researcher (ES) verbally explained the study using the explanatory booklet containing information about the provision of confidentiality and anonymity of their data. The pre-test and post-test were consecutively numbered and anonymously consolidated. The questionnaire was given by the researcher (ES) and the pre-test asked the participants to indicate their consent to the study. A check mark in the consent box was considered to indicate voluntary participation in the study. A pre-test consisting of 4 measurement tools was administered to all eligible women who had agreed to participate during their antenatal check-up after 34 weeks of gestation.

Participants in the control group received only the standard information pamphlet as the usual care from the researcher (ES) after the pre-test. The pamphlet, which consists of 10 pages of A4-sized paper, provides information on the benefits and risks of epidural anesthesia.

Participants in the intervention group received the DA which consists of 22 pages of A4-sized paper from the researcher (ES).”

**Comment 6)**

**Is it possible that the information given (apart from the document) was different between the 2 periods? To get an idea of the reading effort for pregnant women, you may detail le time required to read each document in Japanese (pamphlet and DA).**

**Response:**

Thank you for your important comments. This study did not collect any information from other sources given between the pre-test and post-test.

We have added the time required to read the Japanese DA or pamphlet as follows (Page 9, Lines 159-160):

“It took about 20 minutes for the participants to read the DA or pamphlet.”

**Comment 7)**

**Do the DA describe alternative pain management methods?**

**Response:**

Thank you for your important question. In this particular study, we focused only on either *with epidural anaesthesia* or *without epidural anaesthesia*. We did not include other pain management methods in the DA. However, there have been studies of a DA that included 10 methods of pain relief.

**Comment 8)**

**Why did you choose to measure DSC and satisfaction at one week and not at or just after delivery?**

**Response:**

Thank you for your question. We chose late pregnancy because it can be determined whether or not the pregnant women could undergo vaginal delivery. It was also set at about one week because the pregnant women were required to submit a birth plan at about 36 weeks of gestation. In the future, we would like to investigate the actual method of delivery and satisfaction after delivery.

**Comment 9)**

**Statistical analysis**

**Please precise how you define the study sample.**

**It is not clear in the statistical analysis part that the outcomes will be assessed by differences in score means. It should be specified.**

**Before performing Student t test, have you checked that hypothesizing a normal distribution seemed correct? Please consider justifying that somewhere.**

**Response:**

We truly appreciate your important comments and suggestions. We added a description of the data analysis to define the sample size calculation, assessment of outcomes by differences in mean scores, and normal distribution of data as follows (Page 15, Line 260 -265):

“The data were descriptively analyzed. To determine if the data were normally distributed, a histogram was used to illustrate the distribution. Sample size was calculated from the difference in the mean DCS scores for the primary outcome, and therefore was analyzed by the difference in mean scores. The baseline characteristics and outcomes of the participants were compared between women in the intervention group and women in the control group using the independent t-test or chi-square test.”

**Comment 10)**

**Results and figure**

**A Flowchart should be added with the exact number of eligible women during the period and the number of women non included with the reason. It could also be interesting, if possible, to present as additional data the comparison of eligible women included versus non included to document selection bias.**

**Response:**

Thank you for your important comments and suggestions. We added a flowchart showing the exact number of eligible women and the number of women excluded with a reason (please see **Fig 2** on page 16, Line 286). Selection bias was mentioned in the Limitations section as follows (Page 24, Lines 402-405):

“As this study was not an RCT, bias in the results cannot be completely ruled out. A subsequent RCT is necessary to allow generalization of the results. In the future, we need to consider against selection bias in parity and previous surgery/delivery.”

**Comment 11)**

**In addition to the differences of means, you should present the means score for the pre-test for each group to see if there was baseline difference. For example, how to explain that there seems to be a difference in the rate of indecision (Figure 1), which was more important at baseline in the control versus intervention group? Can we assume that the discourse of caregivers changed in intervention period?**

**Presenting these means would also allow us to know the mean level of knowledge, satisfaction and DCS. If the difference is significant but the overall level is low, the tools remain insufficient.**

**Figure 1: you should detail the percentage rather than the number for clarity.**

**Response**:

We truly appreciate all your important comments to improve the clarity of the manuscript. We presented the mean scores for the pre-test and post-test for each group to allow comparison of the DCS (Please see **Table 2** on page 18), and Satisfaction with decision-making and Knowledge of epidural anesthesia **(**Please see **Table 3** on page 19**)**. We also added comparisons in the revised manuscript as follows:

(Page 17, Line 291-298 DCS)

“**Primary outcome**

The mean score differences in the primary outcomes from pre-test to post-test in the intervention and control groups are shown in **Table 2**. The pre-test showed that the control group had a significantly higher DCS score (0-100) than the intervention group (intervention group: 31.9 [SD 13.5] vs. control group: 36.2 [SD 14.3], p = .009). Women assigned to the intervention group had a significantly lower mean DCS score than women assigned to the control group (intervention group: -8.41 [SD 8.79] vs. control group: -1.69 [SD 5.91], p < .001). The mean score differences in the DCS subscales were significantly lower in the intervention group than in the control group.”

(Page 18, Lines 301-307 regarding ‘Satisfaction with decision-making’)

“**Secondary outcomes**

The mean score differences in the secondary outcomes from pre-test to post-test in the intervention and control groups are shown in **Table 3** and **Fig 3**. The pre-test showed that the intervention group had a significantly higher satisfaction with decision-making score (0-100) than the control group (intervention group: 67.2 [SD 15.2] vs. control group: 62.7 [SD 15.1], p = .013). There was a significant difference in the satisfaction with decision-making score between the 2 groups (intervention group: 9.52 [SD 10.9] vs. control group: 0.76 [SD 6.29], p < .001).”

(Page 18, Line 307-309 regarding ‘Knowledge score of epidural anesthesia’)

“The pre-test showed that the intervention group had a significantly higher knowledge score (0-10) than the control group (intervention group: 7.34 [SD 1.20] vs. control group: 6.59 [SD 1.33], p < .001). The mean score of knowledge of epidural anesthesia was significantly higher in women assigned to the intervention group than in women assigned to the control group (intervention group: 1.96 [SD 1.63] vs. control group: 1.33 [SD 1.44], p <.001).”

As for **Figure 3**, we detailed the percentages for clarity as follows (Page 19, Lines 319-320):

“**Fig 3. Percentage of changes in the decision on using anesthesia: without epidural anesthesia, undecided, or with epidural anesthesia.**”

**Comment 12)**

**Discussion**

**Please discuss why you couldn’t do a randomized controlled trial in the limit section of your manuscript. It could have prevented from comparability bias.**

**Have you checked that your results were not affected by the difference in educational level between control and intervention group by doing sensitivity analysis among high education level women? It may help you to discuss the potential selection bias.**

**Response:**

Thank you for your in-depth comments and important advice. To our knowledge, there is still no study in Japan with regards to the use of a DA for assisting informed decision-making whether to use epidural anesthesia or not. The reasons vary from the small percentage of hospitals that can provide epidural anesthesia, failure to provide information about epidural anesthesia to all pregnant women, to a cultural belief in Japan that labor pain is a virtue. To the best of our knowledge, ours is the first study to do so. However, at the outset, we had existing limitations such that one of the authors belonged to a recruited hospital, there were missing educational data primarily from the control group, difficulty in ascertaining knowledge gained, and the inclusion of both nulliparous and parous women. These hindered the rigorous performance of an exact and strict RCT or would have resulted in an RCT with a number of biases that would have affected the outcomes. We assessed that an **nRCT** at this stage would be sufficient as a pioneering foundation for a subsequent RCT devoid of selection bias in previous parity or delivery or other limitations. We have added an explanation regarding these points in the revised manuscript with an important note for planning an RCT in the future (Page 22, Lines 396-407):

“A limitation in this study is that it is not known if the 2 groups were similar because of missing educational data, which was primarily from the control group. Also, we were not able to ascertain the knowledge gained by the participants from other sources of information (e.g., books, internet) between the pre-test and the post-test. It is important to state that the subjects of this study were limited to Japanese women, as access to analgesia and pain relief differs by country, culture, and medical system. As this study was not an RCT, bias in the results cannot be completely ruled out. A subsequent RCT is necessary to allow generalization of the results. In the future, we need to consider against selection bias in parity and previous surgery/delivery. An assessment as to how a rigorous RCT could be performed is underway as the DA is being planned to be uploaded and viewed on the web rather than in paper form.”

**Journal Requirements:**

**1. Please ensure that your manuscript meets PLOS ONE's style requirements, including those for file naming. The PLOS ONE style templates can be found at**

[**https://journals.plos.org/plosone/s/file?id=wjVg/PLOSOne_formatting_sample_main_body.pdf**](https://journals.plos.org/plosone/s/file?id=wjVg/PLOSOne_formatting_sample_main_body.pdf) **and**[**https://journals.plos.org/plosone/s/file?id=ba62/PLOSOne_formatting_sample_title_authors_affiliations.pdf**](https://journals.plos.org/plosone/s/file?id=ba62/PLOSOne_formatting_sample_title_authors_affiliations.pdf)

**Response:**

We have carefully modified the manuscript in accordance with all the important comments raised and thoroughly checked **PLoS ONE** guidelines to ensure that the revised manuscript meets the style requirements.

**2. Thank you for stating the following in the Funding Section of your manuscript:**

**"This study was supported by grants from the Japan Society for the Promotion of Science (P1-Shigeko Horiuchi 17H01613) and (P2-Naoko Arimori 17H04427)
(**[**https://www.jsps.go.jp/english/e-grants/grants01.html)**](https://www.jsps.go.jp/english/e-grants/grants01.html))**."**

**We note that you have provided funding information that is not currently declared in your Funding Statement. However, funding information should not appear in the Acknowledgments section or other areas of your manuscript. We will only publish funding information present in the Funding Statement section of the online submission form.**

**Please remove any funding-related text from the manuscript and let us know how you would like to update your Funding Statement. Currently, your Funding Statement reads as follows:**

**"No"**

**Response:**

Thank you for your kind reminder. We removed all funding-related text from the manuscript and updated our **Funding Statement section** of the online submission form**.**

“This study was supported by grants from the Japan Society for the Promotion of Science (P1-Shigeko Horiuchi 17H01613) and (P2-Naoko Arimori 17H04427) (<https://www.jsps.go.jp/english/e-grants/grants01.html)>.”]]

**3. Please include your tables as part of your main manuscript and remove the individual files. Please note that supplementary tables (should remain/ be uploaded) as separate "supporting information" files.**

**Response:**

We included the tables as part of the main manuscript and kept the supplementary figures and tables as separate “supporting information” files.

**4. Please upload a copy of Supporting Information Files which you refer to in your text on page 22.**

**Response:**

We uploaded a copy of the Supporting information files we referred to on page 33.
